# Detection of Live Circulating Tumor Cells by a Class of Near-Infrared Heptamethine Carbocyanine Dyes in Patients with Localized and Metastatic Prostate Cancer

**DOI:** 10.1371/journal.pone.0088967

**Published:** 2014-02-14

**Authors:** Chen Shao, Chun-Peng Liao, Peizhen Hu, Chia-Yi Chu, Lei Zhang, Matthew H. T. Bui, Christopher S. Ng, David Y. Josephson, Beatrice Knudsen, Mourad Tighiouart, Hyung L. Kim, Haiyen E. Zhau, Leland W. K. Chung, Ruoxiang Wang, Edwin M. Posadas

**Affiliations:** 1 Uro-Oncolgy Research Laboratories, Samuel Oschin Comprehensive Cancer Institute at Cedars-Sinai Medical Center, Los Angles, California, United States of America; 2 Urologic Oncology Program, Samuel Oschin Comprehensive Cancer Institute at Cedars-Sinai Medical Center, Los Angles, California, United States of America; 3 Division of Hematology Oncology-Department of Medicine, Cedars-Sinai Medical Center, Los Angeles, California, United States of America; 4 Divsion of Urology- Department of Surgery, Cedars-Sinai Medical Center, Los Angeles, California, United States of America; 5 Translational Pathology and Biobank, Cedars-Sinai Medical Center, Los Angeles, California, United States of America; 6 Biostatistics and Bioinformatics Research Center, Cedars-Sinai Medical Center, Los Angeles, California, United States of America; 7 Department of Urology Xijing Hospital, The Fourth Military Medical University, Xi’an, Shaanxi, China; 8 Department of Epidemiology, Xijing Hospital, The Fourth Military Medical University, Xi’an, Shaanxi, China; University of Pécs Medical School, Hungary

## Abstract

Tumor cells are inherently heterogeneous and often exhibit diminished adhesion, resulting in the shedding of tumor cells into the circulation to form circulating tumor cells (CTCs). A fraction of these are live CTCs with potential of metastatic colonization whereas others are at various stages of apoptosis making them likely to be less relevant to understanding the disease. Isolation and characterization of live CTCs may augment information yielded by standard enumeration to help physicians to more accurately establish diagnosis, choose therapy, monitor response, and provide prognosis. We previously reported on a group of near-infrared (NIR) heptamethine carbocyanine dyes that are specifically and actively transported into live cancer cells. In this study, this viable tumor cell-specific behavior was utilized to detect live CTCs in prostate cancer patients. Peripheral blood mononuclear cells (PBMCs) from 40 patients with localized prostate cancer together with 5 patients with metastatic disease were stained with IR-783, the prototype heptamethine cyanine dye. Stained cells were subjected to flow cytometric analysis to identify live (NIR^+^) CTCs from the pool of total CTCs, which were identified by EpCAM staining. In patients with localized tumor, live CTC counts corresponded with total CTC numbers. Higher live CTC counts were seen in patients with larger tumors and those with more aggressive pathologic features including positive margins and/or lymph node invasion. Even higher CTC numbers (live and total) were detected in patients with metastatic disease. Live CTC counts declined when patients were receiving effective treatments, and conversely the counts tended to rise at the time of disease progression. Our study demonstrates the feasibility of applying of this staining technique to identify live CTCs, creating an opportunity for further molecular interrogation of a more biologically relevant CTC population.

## Introduction

Solid tumors are in a constant state of evolution with progressive heterogeneity [1], [2]. The process of metastatic progression is accompanied by multiple phenotypic alternations that result in decreased adhesiveness and increased cellular motility among other alterations [3]. Some motile cancer cells have the capacity to disseminate to distant sites via the vasculature and lymphatic channels and invade tissue leading to formation of a metastatic lesion [4]. Circulating tumor cells (CTCs) thus form a key link between primary tumors and their distant metastases, demarcating irreversible progression of the disease. Isolation and characterization of these live and active cancer cells may improve disease prognosis, as has been demonstrated in prostate cancer (PCa) [5].

The shedding of CTCs is a dynamic process that occurs with both primary and metastatic tumors. The fact that disseminated tumor cells can be detected in the blood of PCa patients after prostatectomy [6] suggests that CTCs can be shed from either residual tumor in the prostate bed or from metastatic deposits. Molecular investigation of these cells may provide real-time information on the status of malignant progression. As the collection of CTCs typically requires low-volume standard phlebotomy, some have proposed that CTCs may be exploited as an ideal surrogate tissue or liquid biopsy to gauge disease status [7]. Such a source of tissue would provide a simple, minimally-invasive tissue source that could be accessed serially to provide high temporal definition of the evolution of underlying disease.

The predictive value of CTCs relies on technical advances to enable reliable detection and isolation. CTCs constitute only a minute fraction of peripheral blood mononuclear cells (PMBCs). Many new technologies are presently being tested for CTC detection and isolation [8]. The most commonly employed strategy relies on epithelial lineage-specific markers such as EpCAM [9] or on size differences relative to PBMCs [10]. The only FDA-approved CTC assay uses an immunomagnetic separation technique based on the expression of epitheial surface markers [11], [12]. The relatively low sensitivity of the assay, coupled with the requirement for pre-fixation makes the isolates unsuitable for molecular analysis beyond immunofluorescence. The dependence on marker expression does not allow for comprehensive detection of the heterogeneous CTC pool. It is also known that not every CTC will result in a new metastatic lesion. The pool of CTCs is composed of live and actively metastasizing cells and bystanders that are passively shed into the circulation [5], [13]–[15], in combination with apoptotic tumor cell debris [16]–[18]. Alternative CTC detection strategies are needed, to isolate the metastasizing fraction, which is most likely to be found in the live CTC pool.

To develop a cost-effective method to identify live CTCs, we assessed the feasibility of using a group of synthetic near infrared (NIR) heptamethine carbocyanine dyes. We have previously demonstrated that these organic dyes are specifically transported into tumor cells and can distinguish malignant from nonmalignant cells in xenograft models or spontaneous tumors *in vivo,* and in surgical tumor specimens *in vivo* or *ex vivo* [19], [20]. These dyes are taken up and accumulated by cancer cells through an active transport system independent of the conventionally employed epithelial surface marker (EpCAM). Furthermore uptake of the dye requires active (ATP-driven) transport and thus can be viewed as a functional assay for tumor cell viability. The results from the current study suggest that IR-783, the prototype of this group of selected NIR dyes, can be incorporated into several detection protocols to identify live CTCs. These live CTCs could be isolated from cancer patients prior to and during therapeutic intervention given the relatively non-invasive means of procurement. Further molecular interrogation could then ensue even at the single-cell level [21].

## Materials and Methods

### Antibodies and Reagents

Fluorescein isothiocyanate (FITC)-conjugated mouse monoclonal antibody (mAb) to human EpCAM (clone 9C4) and phycoerythrin (PE)-conjugated mouse mAb to CD45, together with purified isotype-matched IgG1 and IgG2b, were purchased from BioLegend (San Diego, CA). The source and use of other antibodies has been previously reported, including those against human RANKL, HIF-1α, NRP-1, and VEGF, as well as those to phosphorylated c-MET and the p65 subunit of NFκB [22]. The sources and purification of heptamethine carbocyanine dyes have been reported previously [19], [20]. Ficoll-Paque PREMIUM 1.084 was purchased from GE Healthcare (Piscataway, NJ), and Histopaque-1077 was purchased from Sigma-Aldrich (St. Louis, MO).

### Cell Culture

Human PC-3 cells were obtained from American Type Culture Collection (Manassas, VA). The cells were cultured in RPMI-1640 medium supplemented with 5% fetal bovine serum (FBS, Gemini Bio-Products, West Sacramento, CA), penicillin (100 unit/ml) and streptomycin (100 µg/ml). Cells were detached with 0.05% trypsin/EDTA (Invitrogen, Carlsbad, CA), washed in Ca^2+^- and Mg^2+^-free phosphate buffered saline (PBS, Invitrogen), and resuspended in complete culture medium. Cells were counted on a TC10 Automated Cell Counter (Bio-Rad, Hercules, CA), with trypan blue exclusion to confirm cell viability.

### Clinical Blood Specimens

Patient blood samples were used with written informed consent through Cedars-Sinai Medical Center institutional review board-approved bio-banking protocols (IRB #Pro00025217 and #Pro00030418). Clinical blood samples were collected pre-operatively from 40 PCa patients undergoing radical prostatectomy at Cedars-Sinai Medical Center. Multiple blood samples were obtained from repeated visits of 5 patients with androgen independent disease. Additional blood samples from 34 healthy male donors aged between 32 and 70 years were obtained. About 7.5 ml of venous blood was collected into an EDTA-containing lavender top tube (BD, Franklin Lakes, NJ), and centrifuged at 1,500 rpm for 20 minutes at room temperature on a Heraeus CLINIFUGE centrifuge (Thermo Scientific, Logan, UT). After the plasma fraction was harvested for diagnostic purposes, the packed blood cell fraction was transported on ice to the research laboratory within 3 hours of collection.

### Isolation of PBMCs

PBMCs were isolated with standard density gradient centrifugation. The packed blood sample was diluted with an equal volume of a balanced salt solution containing 0.01% glucose (w/v), 5 µM CaCl_2_, 9.8 µM MgCl_2_, 540 µM KCl, 126 mM NaCl, and 14.5 mM Tris, pH 7.6. A 2-ml aliquot of the diluted sample was layered onto a 3 ml Ficoll-Paque cushion and subjected to centrifugation at 400×g at room temperature for 40 minutes. Cells in the nucleated cell fraction were collected and washed twice in PBS, and resuspended in RPMI-1640 medium containing 5% FBS for further analyses.

### Tumor Cell Spiking and Staining

Aliquots of the PBMCs were made so that each contained an equivalent number of cells from 1 ml donor blood, about 2×10^6^ cells/aliquot. To spike with cancer cells, known numbers of live PC-3 cells in RPMI 1640 medium containing 5% FBS were added to the PBMC aliquot. In control studies spiked with dead cancer cells, PC-3 cells in single cell suspension were killed in 75% ethanol and recovered in the same medium. The spiked samples were then stained with 20 µM NIR dyes at 37°C for 30 minutes. After washing twice in PBS to remove free dyes, the cells were fixed in formalin for 10 minutes at room temperature, washed twice in PBS, and resuspended in 400 µl Cell Staining Buffer (BioLegend). To further stain for surface markers, the samples were incubated first with isotype IgG on ice for 10 minutes, and then reacted simultaneously with FITC-conjugated mAb to EpCAM and PE-conjugated mAb to CD45 for 20 minutes. The working ratio of the mAbs was 0.1 µg/10^6^ cells. After washing twice with the Cell Staining Buffer, the cells were stained with 4′,6′-diamidino-2-phenylindole (DAPI, Invitrogen) before being subjected to detection.

### Detection of CTC with Fluorescence Activated Cell Sorting (FACS)

Two FACS instruments (BD Biosciences, MA) were used in the study. To isolate CTCs, a FACSAria III was used to sort positively labeled cells onto an APES-coated cytology slide (Bio-World, Dublin, OH). To enumerate CTCs, an LSRII Flow Cytometer was used. Manufacturer recommended detection procedures were followed. In parallel to the detection of each human blood sample, 1×10^4^ PC-3 cells were used to spike a 1 ml aliquot of the sample. The flow cytometric profile of the spiked sample was used to guide the positivity gating. FACS data was further analyzed with FlowJo software.

### Fluorescence Imaging

Stained cells isolated with the FACS sorter on slides were subjected to both fluorescence imaging and near infrared imaging with our previously reported procedure [19], [20], [22], with a Nikon Eclipse Ti fluorescence microscope excited by a xenon arc light source. Near infrared images were acquired through an INDO filter (780–840 nm).

### Multiple Quantum Dot Labeling (mQDL)

Stained cells collected with the FACS sorter on glass slides were subjected to further staining with the mQDL protocol as previously reported [22]. In brief, the samples were first treated with stripping buffer to remove the mAb used for CTC isolation, and then subjected to successive staining with antibodies reacting to a group of PCa-related biomarkers, including RANKL, HIF-1α, NRP-1, VEGF, p-c-MET, and p-p65, as previously reported [22], with the same staining protocol. Finally, the samples were counterstained with DAPI before being subjected to spectral imaging and signal quantification on a CRi spectral imaging system with Nuance software (Caliper Life Sciences, Hopkinton, MA).

### Statistical Analysis

Means, standard deviations, and medians were used to summarize continuous variables. Frequencies and percentages were calculated for categorical variables. Spearman correlations were calculated between continuous variables in the data due to skewed distributions. Comparisons of live CTC between categories of interest were done using Wilcoxon Rank Sum tests. A linear mixed model was used to assess the relationship between the number of cells spiked and retrieved, where the repeated measures on the donors were modeled with a heterogeneous compound symmetric covariance structure. All analyses were done using SAS version 9.3.

## Results

We have previously demonstrated active uptake and retention of NIR dyes by cultured human PCa LNCaP, C4-2, PC-3, DU-145, ARCaP_E_ and ARCaP_M_ cells [19], [20]. As few as ten PC-3 cells spiked into 1 ml human donor blood can be detected using IR-783. Dead PC-3 cells spiked in the same fashion failed to exhibit any IR-783 uptake. Because uptake of IR-783 requires the use of an ATP-requiring organic anion transporter, dye uptake itself is a functional assay which points toward tumor cell viability. To assess applicability of this approach to detecting live CTCs in patient blood samples, we incorporated NIR dye staining into FACS analysis. The widely used EpCAM^+^CD45^−^profile [9], [12] was adopted to consolidate the CTC nature of the detected NIR^+^ cells.

### 1. Identification and Isolation of Live (NIR^+^) Cells from Human Blood

Patient blood samples were subjected to gradient centrifugation then sequential staining: first with NIR dye to stain live CTCs, then with fluorescence-labeled antibodies to EpCAM and CD45 for confirmation. Finally, the sample was stained with DAPI for the visualization of cell nuclei.

**Figure 1:**
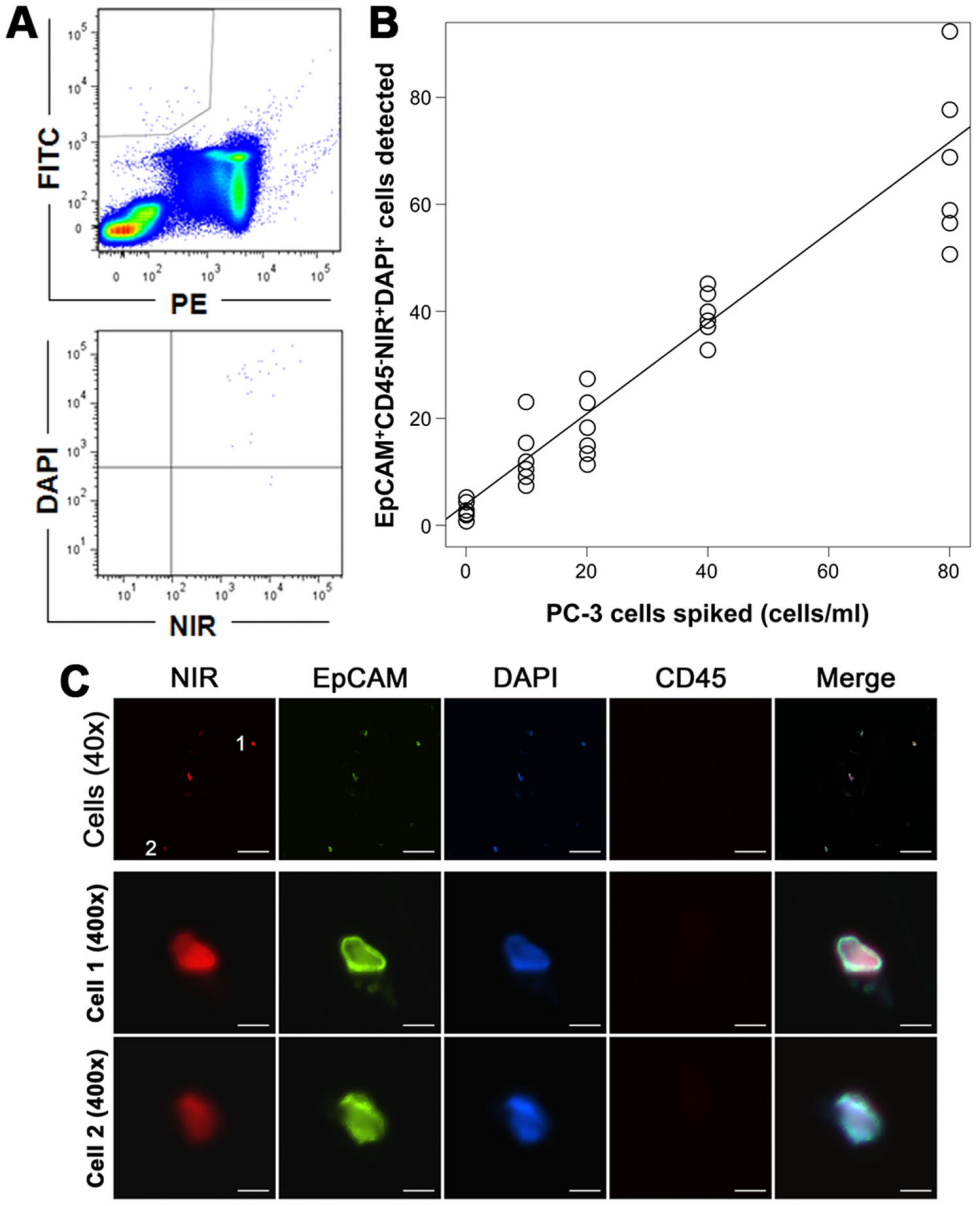
Calibrating the CTC detection method. Human PCa PC-3 cells were used as positive control cells. **A**, PC-3 cells stained sequentially with all four detection agents were subjected to FACS analysis. Upper panel: the sample was first detected for positive EpCaM (FITC) but negative CD45 (PE) stain (gated fraction). Lower panel: the gated fraction was queried for IR-783 (NIR) and nuclear (DAPI) stain. NIR^+^EpCAM^+^DAPI^+^CD45^−^ counts were considered as live cancer cells. PC-3 detection was used in parallel in the analysis of each clinical specimen as guidance in gate setting. **B**, Efficiency of the detection strategy was assessed by comparing the number of spiked and detected PC-3 cells by FACS analysis. Each data point was the mean of detected PC-3 cells spiked to 6 healthy donor samples. **C**, Fluorescence microscopy was used to visualize PC-3 cells recovered onto a glass slide. Upper row shows the images of a representative field at low magnification (40×; bar = 250 µm), with two cells (marked with 1 and 2) presented at high magnification in the lower two rows (400×; bar = 25 µm).

As a control to calibrate the performance of the detection system, we first tested the staining protocol with healthy donor blood spiked with PC-3 cells at varying numbers. In FACS analysis, the stained samples were sorted to isolate the EpCAM^+^CD45^−^ population. These cells were then resorted to determine the number of NIR^+^DAPI^+^ cells (Figure 1A). PC-3 cells were identified as nucleated cells (DAPI^+^) expressing EpCAM but not CD45 (Figure S1). In repeated tests when the final EpCAM^+^CD45^−^NIR^+^DAPI^+^ counts were modeled as a function of spiked cell number, a significant increasing trend was observed with a correlation coefficient of 0.997 (Figure 1B and Table S1). The highly linear correlation suggested that this FACS protocol was usable for detecting CTCs with a negligible number of non-specific counts. We further determined that the recovered PC-3 cells on microscope slides could be readily seen by fluorescence microscopy (Figure 1C). Using the same protocol, we detected 2.8±1.7 EpCAM^+^CD45^−^NIR^+^DAPI^+^ events in a series of studies with blood specimens collected from 34 healthy donor subjects (Figure 1B). This detection was well in agreement with other studies on EpCAM^+^ cells in healthy donor blood samples analyzed using next generation CTC platforms [23], and probably reflects the background level of circulating epithelial cells. Importantly, in parallel studies where dead PC-3 cells were used in spiking, the EpCAM^+^CD45^−^NIR^+^DAPI^+^ counts were maintained at the background level, suggesting that the spiked dead PC-3 cells did not pick up the dye (data not shown). These control studies collectively demonstrate the suitability of NIR dye to stain and detect live CTCs.

### 2. Detection of Live CTCs in Patients with Localized PCa Undergoing Radical Prostatectomy

Using the functional FACS assay following NIR staining, we tested the detection of EpCAM^+^CD45^−^DAPI^+^ cells, henceforward referred to as *total* CTCs, in clinical PCa patients with emphasis on the EpCAM^+^CD45^−^NIR^+^DAPI^+^ events (henceforward referred to as *live* or *NIR^+^* CTCs). PBMCs were isolated from pre-operative blood samples of 40 patients diagnosed with localized diseases. Detailed characteristics of these patients are summarized in Table 1.

**Table 1:** Detailed summary of the patients undergoing prostatectomy.

Patient ID	GleasonScore	T stage	N stage	Marginstatus	Stephensonprediction	Pre-operation PSA(ng/ml)	Post-operation PSA(ng/ml)	Total CTC(counts/ml)	NIR^+^ CTC(counts/ml)	Live CTC(%)
1	3+3	pT2a	pN0	**–**	98%	4.76	<0.1	8	4	50%
2	3+4	pT2c	pN0	**+**	95%	2.7	<0.1	4	2	50%
3	3+4	pT2c	pN0	**–**	98%	1.9	<0.1	15	10	67%
4	3+4	pT2c	pN0	**–**	96%	4.6	<0.1	26	19	73%
5	3+4	pT2c	pNx	**+**	92%	8.34	<0.1	65	7	11%
6	3+3	pT2c	pN0	**–**	98%	4.5	<0.1	10	10	100%
7	3+3	pT2c	pN0	**+**	97%	4.33	<0.1	12	10	83%
8	3+4	pT2c	pN0	**–**	96%	4.54	<0.1	34	8	24%
9	3+4	pT2c	pNx	**–**	96%	5.2	<0.1	10	10	100%
10	4+3	pT2c	pN0	**–**	89%	6.96	<0.1	4	4	100%
11	3+3	pT2c	pNx	**–**	97%	9.16	<0.1	1	1	100%
12	3+4	pT2c	pN0	**–**	96%	4.9	<0.1	2	2	100%
13	3+4	pT2c	pN0	**+**	93%	5.9	<0.1	8	5	63%
14	3+4	pT2c	pN0	**–**	96%	5	<0.1	151	148	98%
15	3+3	pT2c	pN0	**–**	98%	1.53	<0.1	12	11	92%
16	3+4	pT2c	pN0	**–**	95%	7.69	<0.1	5	5	100%
17	3+3	pT2c	pNx	**+**	97%	4.35	<0.1	8	8	100%
18	3+3	pT2c	pN0	**+**	96%	5	<0.1	10	10	100%
19	3+4	pT2c	pN0	**–**	94%	14.02	<0.1	21	20	95%
20	3+4	pT2c	pN0	**–**	95%	7	<0.1	23	13	57%
21	3+3	pT2c	pN0	**–**	97%	7.3	<0.1	54	53	98%
22	3+4	pT2c	pN0	**+**	95%	2.79	<0.1	117	116	99%
23	3+4	pT2c	pN0	**–**	95%	5.7	<0.1	0	0	
24	3+3	pT2c	pN0	**–**	96%	16.44	<0.1	26	26	100%
25	3+4	pT2c	pNx	**+**	96%	4.26	<0.1	6	5	83%
26	3+3	pT2c	pN0	**–**	98%	4.02	0.14	3	3	100%
27	3+4	pT3a	pN0	**+**	85%	5	<0.1	119	87	73%
28	3+4	pT3a	pN0	**–**	89%	6.1	<0.1	16	11	69%
29	4+5	pT3a	pN0	**+**	34%	33	0.43	57	46	81%
30	4+3	pT3a	pN0	**–**	79%	4.85	<0.1	14	13	93%
31	3+4	pT3a	pN0	**–**	90%	4.2	<0.1	53	48	91%
32	3+4	pT3a	pNx	**+**	84%	6.09	<0.1	22	10	45%
33	4+3	pT3a	pNx	**–**	78%	5.5	<0.1	141	132	94%
34	3+4	pT3a	pN0	**–**	87%	9.95	<0.1	8	7	88%
35	3+4	pT3a	pN0	**–**	89%	5.6	<0.1	8	8	100%
36	4+3	pT3a	pN0	**–**	77%	6.05	<0.1	46	43	93%
37	3+4	pT3a	pN0	**+**	86%	4.3	<0.1	26	19	73%
38	4+3	pT3b	pN0	**+**	32%	10	<0.1	8	7	88%
39	4+3	pT3b	pN1	**+**	15%	12.6	0.2	440	439	100%
40	3+3	pTx	pN0	**+**	N/A	9.67	<0.1	54	48	89%

**Figure 2:**
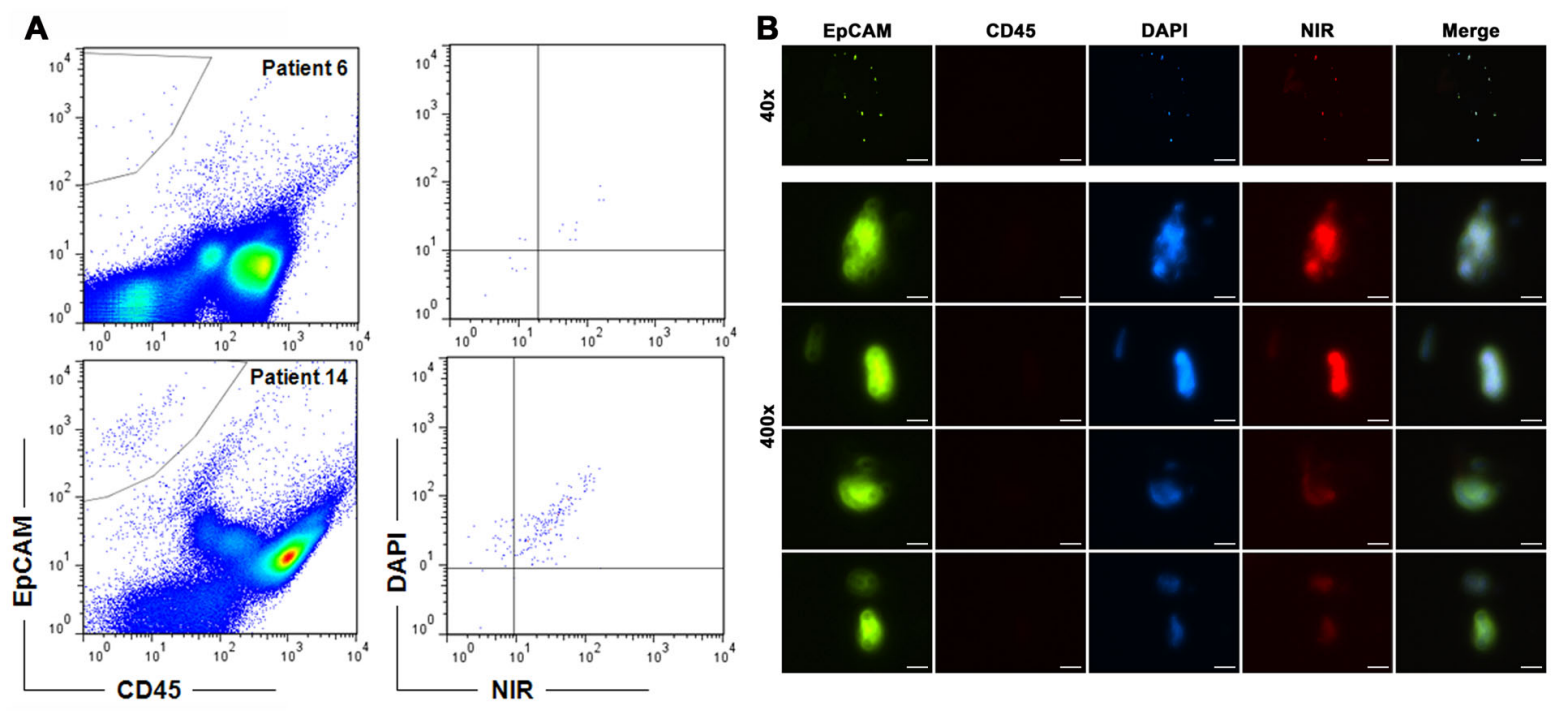
Application of the detection protocol to clinical samples from PCa patients. Representative detection results are shown. **A**, upper row shows CTC detection from patient 6, where 10 live CTCs were found. Lower row shows the detection of 148 live CTCs from patient 14. For each sample, gate setting was directed by parallel staining of PC-3 cells. **B**, CTCs detected from patient 14 were isolated onto a microscope slide and visualized immediately by fluorescence imaging. Low magnification images of a representative field (40×; bar = 250 µm) are shown in the upper row. Below, high magnification images of 2 live CTCs (NIR^+^) from the same field are shown in the next two rows, in comparison to 2 dead CTCs (NIR^−^) in the last two rows (400×; bar = 25 µm).

This series of analysis revealed that live CTC counts varied remarkably among patients. EpCAM^+^CD45^−^NIR^+^DAPI^+^ counts ranged from 0 to 439 cells/ml (average 25 cells/ml; median 10 cells/ml) for this cohort of perioperative patients (Table 1). In this study, a count of less than 5 was considered normal, as our healthy donor pool had 2.8±1.7 EpCAM^+^CD45^−^NIR^+^DAPI^+^ counts (Figure 1B). The staining method reproducibly detected more than 5 live CTCs/ml in 30 of the 40 patients. Representative results from flow cytometric detection are shown in Figure 2A. We further demonstrated that the CTCs could be isolated directly onto microscope slides, and could be visualized with fluorescence microscopy (Figure 2B). In these analyses, live CTCs were distinguished from dead cells based on uptake of NIR dye (Figure 2B).

The live CTC counts detected from this cohort did not correlate significantly with pre-operative serum PSA level (r = 0.12, *p* = 0.47, Figure S2), pathologic N-stage (given only 1 node positive patient), Gleason score, or margin status (Figure S3). Comparing CTC counts to the Stephenson predictive monogram, there was no statistically significant relationship between live CTC counts and the Stephenson predictive monogram.

There were 10 patients with 5 or fewer live CTCs/ml (Table 1), and all had T2 disease with negative surgical margins. Only one of these patients (patient 26) had a detectable serum PSA concentration 2 weeks after radical prostatectomy. His serum PSA became undetectable at 6 weeks following the operation. The patient has since remained relapse-free. There were 3 patients with positive surgical margins (patients 2, 13, and 25, Table 1). These patients have also remained relapse-free for more than two years so far without additional anti-cancer therapies.

There were 3 patients had detectable serum PSA concentrations within 3 months after radical prostatectomy (Table 1). Patient 26 had a pT2cN0 Gleason 3+3 cancer with negative margins. Interestingly, he had only 3 live CTCs/ml blood. Serum PSA in this patient became undetectable later without additional intervention. Patient 29 had a pT3aN0 Gleason 4+5 cancer with positive margins, and was detected with 46 live CTCs/ml. His serum PSA level became undetectable after 6 months of androgen deprivation therapy that continues until this time. Patient 39 had lymph node metastasis at the time of surgery and was found to have high pre-operative CTC counts (439/ml). Additional cases of recurrence have to be examined in order to determine if pre-operation CTC counts could be used as a parameter for predicting disease relapse.

We also compared live CTC counts to surgical margin status. The 25 patients with negative margins had an average of 25 live CTCs/ml (range 0–148), in comparison to the 15 patients with positive margins that had an average of 54 live CTCs/ml (range 2–439). The difference was not statistically significant, likely due to the marked variation between individuals and the limited sample size.

While most of the patients in our cohort had Gleason 6 or 7 pathologies, no correlation was found between Gleason score and live CTC numbers. On the other hand, patients with higher live CTC counts trended toward having more advanced disease by tumor staging. There was a significant association between live CTC counts and T stage, with pT3 patients having higher counts than pT2 cases (median 8 vs. 19, *p* = 0.02). Notably, the only patient with node-positive pN1 disease had the highest live CTC count (439 CTCs/ml, patient 39, Table 1). High live CTC counts, therefore, seemed correlated to advanced tumor stages.

### 3. Assessments of Live CTCs in Patients with Metastatic Diseases

**Figure 3:**
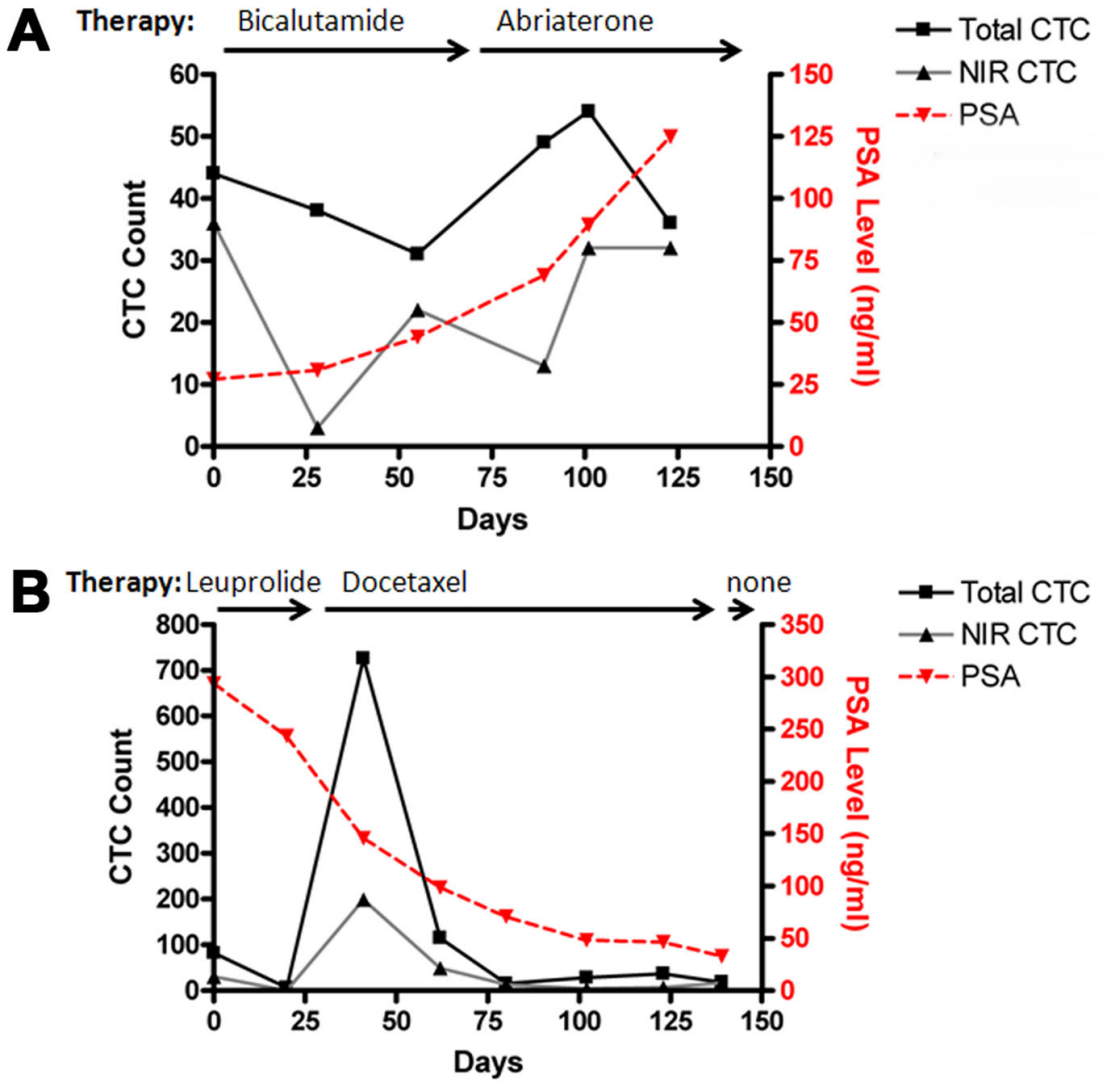
Live CTC counts as an independent indicator of disease progression. CTC counts detected from mCRPC patients were analyzed along with the progress of therapeutic intervention. Two representative results are shown. **A**, the progressive increment of CTC counts in patient 41 correlated with clinical metastatic progression. **B**, the persistent low CTC counts in patient 42 correlated with beneficial anticancer therapies.

Consecutive blood samples were obtained from 5 PCa patients whose disease had relapsed and metastasized following initial therapy. These patients were undergoing various types of systemic treatment. In contrast to the live and total CTC counts in patients with localized disease, greater disparity between total and live CTC counts was seen (Table 2), while CTC counts across cases showed little correlation to serum PSA concentration (Figure 3). Nonetheless, when analyzed over time for individual patients, we identified interesting trends in which live CTC counts seemed to correlate inversely to the observed clinical benefit in response to therapies.

**Table 2:** Summary of metastatic prostate cancer patients.

Patient ID	Day ofanalysis	Clinical information	Therapy	PSA (ng/ml)	Total CTC/ml	NIR^+^ CTC/ml	Live CTC (%)
41	0		Starting bicalutamide	27.1	44	36	82%
	28			30.7	38	3	8%
	55		Starting abiraterone	44.1	31	22	71%
	89			68.9	49	13	27%
	101			89.3	54	32	59%
	123	Worsening bone pain	Starting radiation	124.8	36	32	89%
42	0		Starting docetaxel	293.7	82	30	37%
	20	Improved overall constitution		243.3	6	0	0%
	41			145.7	726	199	27%
	62			98.5	115	49	43%
	80			70.8	15	13	87%
	102			47.9	28	4	14%
	123			46.1	37	7	19%
	139		Ending docetaxel	32.8	18	17	94%
43	0		Starting docetaxel	46.4	36	5	14%
	36	Progressive bone pain		31	44	1	2%
	51	Systemic deterioration	Ending docetaxel	31.8	39	38	97%
44	0		On bicalutamide	3.3	1052	922	88%
	25		Stopping bicalutamide	7.3	54	35	65%
	73	Developing shoulder pain	Starting DES	8.5	196	123	63%
45	0		On leuprolide for 6 months	0.1	1	0	0%
	27			<0.1	6	5	83%
	55	PSA 0.2 ng/ml in one month		<0.1	207	174	84%

Patient 41 began bicalutamide treatment at the start of sample collection for metastatic castration-resistant PCa (mCRPC). He experienced asymptomatic biochemical (*i.e.,* serum PSA) progression for two months, during which live CTC counts initially decreased but rebounded quickly. The treatment was changed to abiraterone acetate, which failed to produce a biochemical response. The patient developed symptomatic osseous metastatic disease and required radiation therapy. During disease progression, total CTC counts were sustained, but the live CTC proportion increased along with serum PSA level (Table 2 and Figure 3A).

Patient 42 was subjected to CTC detection before the first cycle of docetaxel therapy, which resulted in an excellent response judged by the continued drop of serum PSA level even after the therapy was ended. The patient remained asymptomatic for another 4 months before having to undertake another 8 cycles of docetaxel for symptomatic progression. During the treatment, the patient’s CTC counts decreased markedly, from 82 down to 6, and live CTCs decreased accordingly, from 37 to 0%. This patient experienced a rebound in total and live CTCs during the course of docetaxel treatment from day-20 to day-139. Toward the end of docetaxel treatment (from day-102 to day-139), we observed live CTCs rebounding from 14 to 94% (Table 2 and Figure 3B).

Patient 43 initially received docetaxel therapy on which he displayed initial biochemical and clinical responses. The second cycle of docetaxel treatment, however, was followed by rapid disease progression with progressive clinical deterioration (*i.e.*, worsening pain and weight loss) and a rise in serum PSA. We noted a dramatic elevation in the percentage of live CTCs (from 2% up to 97%) without substantial changes of total CTCs (ranged from 36 to 44). The patient expired over the next 2 months with rapid clinical deterioration. The live CTC count rose markedly along with the deterioration (Table 2).

Patient 44 was treated with bicalutamide for mCRPC. The treatment did not result in biochemical benefit but a precipitous drop in CTC counts from 1052 to 54 was noted. Despite this dramatic drop in total CTCs, however, the percentage of live CTCs did not change substantially remaining in the range of 63 to 88%. The CTC counts rose from 54 to 196 between day-25 to day-73 at the time when bicalutamide treatment was terminated. This patient required palliative radiation and a quaternary hormonal maneuver (Table 2).

Patient 45 had castration sensitive PCa and was subjected to intermittent androgen suppression therapy that had been initiated 4 months before the first CTC detection. Live CTC count was below the threshold of detection, in good agreement with the well-suppressed serum PSA concentration (Table 2). In this case, the subsequent rise of live CTC counts was 4 months earlier than the PSA rebound, which was later suppressed with androgen deprivation therapy. It is worth noting, however, that this patient had a high percentage of live CTCs (83–84%) despite hormonal suppression therapy.

### 4. Isolation of Live CTCs for further Molecular Characterization

**Figure 4:**
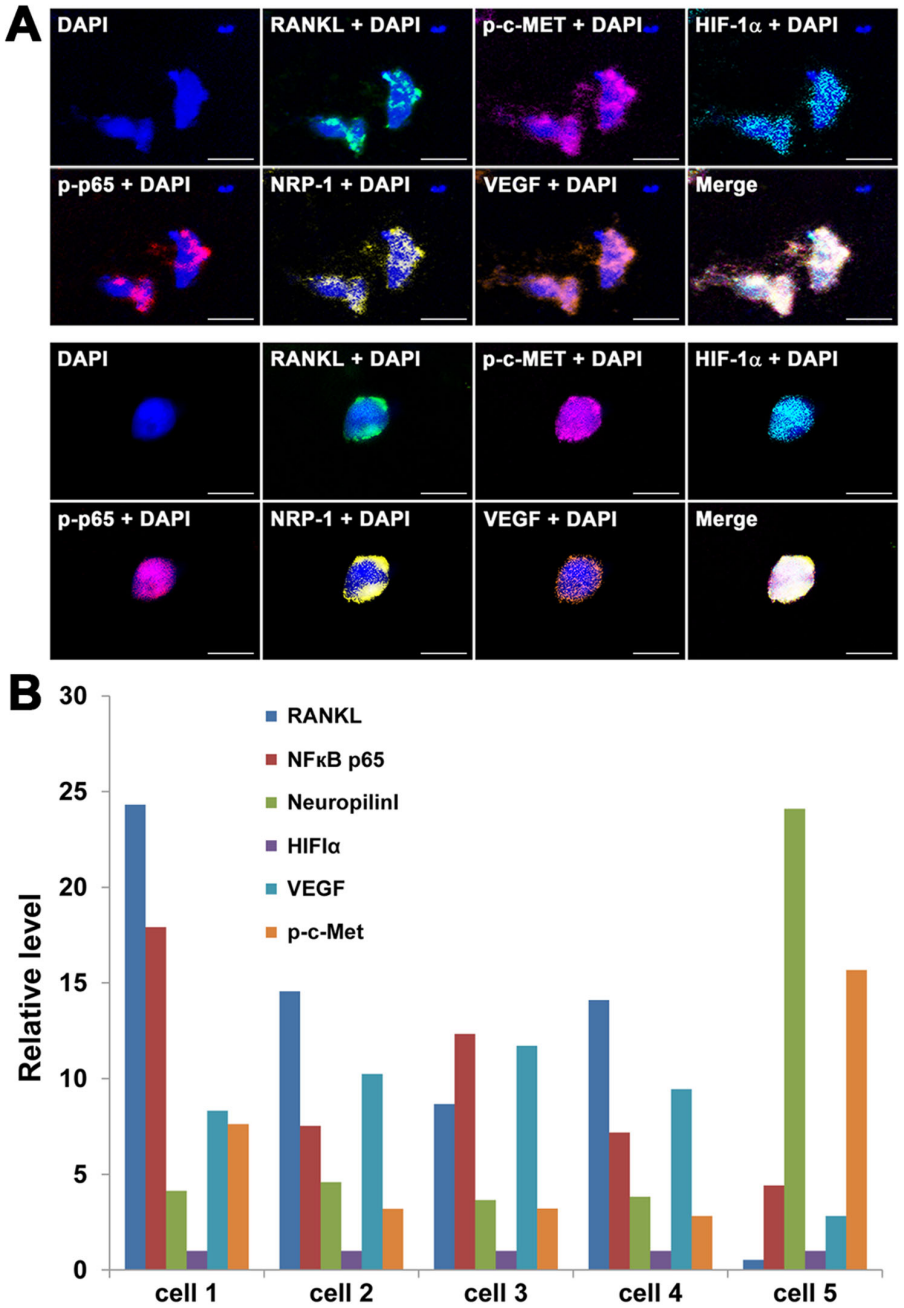
Isolating CTCs for further molecular characterization. Live CTCs from the first sample from patient 44 (Table 2) were isolated onto a microscope slide and subjected to mQDL staining for the status of a panel of proteins documented to relate to PCa progression. **A**, Spectral images from two representative CTCs are shown on the top two panels (8 images each that represent the expression level of RANKL, pc-Met, HIF-1α, pp65, NRP-1 and VEGF; 400×; bar = 50 µm). **B**, Quantification of spectral image intensities of the six proteins indicated in Panel A from five stained cells from the same patient. The relative level of gene expression was calculated based on the expression of HIF-1α which was assigned as 1.0.

NIR staining facilitated the identification and isolation of live CTCs from clinical blood samples (Figure 2). We tested the isolated CTCs to further investigate PCa-related molecular alterations. In one such investigation, CTCs in PCa patients were isolated based on EpCAM^+^CD45^−^NIR^+^DAPI^+^ staining. Protein expression in the CTCs was detected by mQDL and was performed to verify expression of a panel of protein biomarkers associated with PCa progression and metastasis that our group is actively studying [22]. These assays demonstrated that the abnormal expression of RANKL, HIF-1α, NRP-1 and VEGF proteins seen in clinical PCa tumor specimens [22] could be easily detected in the isolated CTCs (Figure 4A). Similarly to clinical tumors, enhanced phosphorylation of c-Met, as well as the p65 subunit of the NFκB, was detected in the same CTC population. Intriguingly, signal quantification of the stained CTCs revealed remarkable intercellular heterogeneity, as individual proteins were detected with varied levels among CTCs (Figure 4B). Further investigation, however, is needed to assess the extent of CTC heterogeneity at the gene expression level.

## Discussion

We developed a protocol for assessing the presence of live CTCs based on the active uptake of prototype IR-783 heptamethine carbocyanine dye [19], [20]. In combination with antibodies to EpCAM, IR-783 staining identified live human PC-3 cells spiked into healthy donor blood with high reproducibility and correlation coefficient (Figure 1). We have determined that active transport of IR-783 into tumor cells can be used as a functional assay to demonstrate tumor cell viability (Figure 2B). This finding is further emphasized by the lack of IR-783 dye uptake in dead tumor cells (freeze-thaw and/or ethanol fixed) spiked into healthy donor blood (data not shown). We successfully detected and isolated live CTCs from clinical samples of primary PCa (Figure 2 and Table 1) and mCRPC patients (Figures 3 and Table 2). In a proof-of-concept study, the isolated live CTCs were amenable to multiplex detection of protein levels at the single cell level in freshly isolated CTCs using an mQDL method (Figure 4). The results collectively suggest that: 1) IR-783 staining can differentiate live CTCs from a total enumerated CTC population in a given patient in real time; 2) Correlations between the percentage of live CTCs and the development of progressive and therapeutic-resistant disease may be established; and 3) Isolated CTCs are appropriate for biological analysis such as mQDL analysis for protein expression at the single cell level. Our results provide for the first time a novel way of characterizing and even enumerating live CTCs in freshly harvested patient blood. This approach allowed us still to use techniques to characterize these live cells by quantifying protein expression in isolated CTCs.

Detection and characterization of CTCs have been technically challenging [24]–[26]. Conventional strategies have used either surface marker expression or cell size. At this time, new detection approaches are in development. EpCAM-decorated buoyant immune-microbubbles, for example, have been used to detect both spiked and endogenous cancer cells from whole blood [27], while cytokeratin has been tested as markers for CTC detection individually [28], or in combination with the EpCAM protein [29]. None of these approaches provides any information on the viability of the CTCs. Although the current FDA-approved detection platform can detect some CTCs [30], we and others have found that this approach relatively insensitive and is unable to detect all the inherently heterogeneous CTCs [31], [32]. Using IR-783 dye staining may circumvent heterogeneity of surface maker expression on tumor cells by using a functional assay to detect all the viable CTCs. CTC apoptosis is a common phenomenon [16]–[18]. We previously determined that only live cancer cells were capable of internalizing and accumulating the class of IR-783 analogs, most likely through the organic anion transporting peptides (OATPs) as carrier proteins [19], [20], [33]–[36]. Application of the NIR dye in CTC detection provides a simple strategy for determining cell viability. In the current study, many of the EpCAM^+^CD45^−^ counts could be anucleated as indicated by the lack of DAPI stain (Figure 2A), likely representing cell debris. Although live and total CTC counts seemed close in perioperative samples, the live CTC counts detected from many mCRPC samples were significantly less than the corresponding total counts (Tables 1 and 2), indicating increased death of CTCs in mCRPC cases undergoing anti-tumor therapy. In this regard, this study has established a cost-effective protocol for total and live CTC detection by direct staining of the cells in combination with rapid FACS detection. This protocol shows potential for evaluating the cytotoxic effects of anti-tumor reagents in real time, during the clinical treatment process.

When different patients were statistically analyzed as a group, this study did not detect a direct correlation between CTC counts and disease progression. Interestingly, lacking of correlation was also reported in another study on different stages of prostate cancers, with an FDA-approved detection method [37]. An important finding of the study is the uniqueness of the CTC profile in individual patients. This is supported by the individual relationships between the CTCs detected and the disease status diagnosed in each patient (Table 1) and by the consistency between the CTCs detected using the protocol and the therapeutic response observed by the attending clinicians (Table 2). CTCs represent a pool of specific types of cancer cells capable of surviving in the blood stream despite the remarkable shear forces. The total number of CTCs at any given time could represent the dynamic equilibrium between tumor shedding and the clearance of CTCs by apoptotic death. Live CTCs, by contrast, could provide crucial information on resistance to therapy and to physical stress during circulation. Live CTCs were found to fluctuate particularly in mCRPC patients and changes can be seen prior to PSA changes in patients (Figure 3 and Table 2). Moreover, the population of CTCs generally shifted toward a greater portion of live cells when systemic therapy was stopped (Table 2). These observations strongly support the usefulness of live CTC to monitor therapeutic efficacy. Presently, most of these patient subjects are still under the care of the oncologist (EP) and urologists (MHTB, CSG, DYJ and HLK), and data on their clinical course continues to be collected. Follow-up data will be evaluated for the applicability of NIR dye-assisted live CTC detection.

The prototype NIR dye IR-783 is a promising agent for live CTC detection. Compared with the EpCAM-based method, IR-783 stain alone was frequently found to be able to detect more candidate CTCs from the same patient sample (data not shown). This finding is consistent with the problem that currently utilized EpCAM-based methods offer incomplete detection and do not capture the entire CTC population. As an initial study to evaluate applicability of the IR-783 dye for CTC detection, we resorted to vestigial EpCAM expression as a reference to confirm the epithelial origin of the IR-783 stained cells, since there were no surface markers specific for CTCs. It is possible that a significant fraction of tumor cells may undergo extensive morphologic and behavioral changes to become migratory and invasive through epithelial to mesenchymal transition, resulting in CTCs with marked suppression or loss of EpCAM expression. Conversely, it is also possible that certain PBMCs in PCa patients have acquired the capacity for NIR dye uptake and retention, and the abnormal NIR staining of patient PBMCs is a sign of cancer progression. Additional investigation is warranted to identify the nature of NIR^+^ cells in patient blood.

In summary, we have established a novel method for identifying live CTCs from clinical samples. This method was demonstrated to be rapid, sensitive and highly efficient in distinguishing live CTCs, which are shown to be changing dynamically in response to therapeutic intervention. Compared to other diagnostic parameters such as serum PSA, alterations in total CTCs and percentage of live CTCs, coupled with the feasibility of further molecular characterization at a single cell level, may provide new insights into the therapeutic response and stress resistance of the disease. Further modification of these methods for high-throughput capability will transform the protocol into a cost-effective detection method that has diagnostic, prognostic and treatment value for PCa patients.

## Supporting Information

Figure S1 Confirmative staining of PC-3 cells with individual detection agents used in this study. A representative stain is shown. **A,** PC-3 cells stained with each individual detection agent were subjected to FACS analysis. In upper row, PC-3 cells stained by a mixture of mouse IgG1-FITC and IgG2b-PE were used as control. In the lower row, PC-3 cells in PBS were used as control. **B,** PC-3 cells were stained first with IR-783, then simultaneously with FITC-labeled antibody to EpCAM and PE-labeled antibody to CD45, and finally stained with DAPI. The stained cells were first detected for EpCAM expression and for CD45 exclusion (upper panel), and then for NIR and DAPI staining (power panel).
(TIF)

Figure S2 Lack of statistical significance when CTC counts from different patients were pooled for correlation analyses. Counts of the candidate CTCs from 40 primary prostate cancer patients and 23 samples from 5 mCRPC cases were analyzed against serum PSA level.
(TIF)

Figure S3 Lack of statistical significance between CTC counts and common diagnostic parameters. Counts of the candidate CTCs from 40 primary prostate cancer patients and 23 samples from 5 mCRPC cases were analyzed against surgical margin, Gleason score, T-stage and N-stage of the patient.
(TIF)

Table S1 Detection of PC-3 cells spiked to donor blood.
(TIF)
