# Supplementary figures and images for: Detection of Live Circulating Tumor Cells by a Class of Near-Infrared Heptamethine Carbocyanine Dyes in Patients with Localized and Metastatic Prostate Cancer
Source: PLoS One. 2014 Feb 14;9(2):e88967. doi: 10.1371/journal.pone.0088967 (PMC3925210; doi:10.1371/journal.pone.0088967)

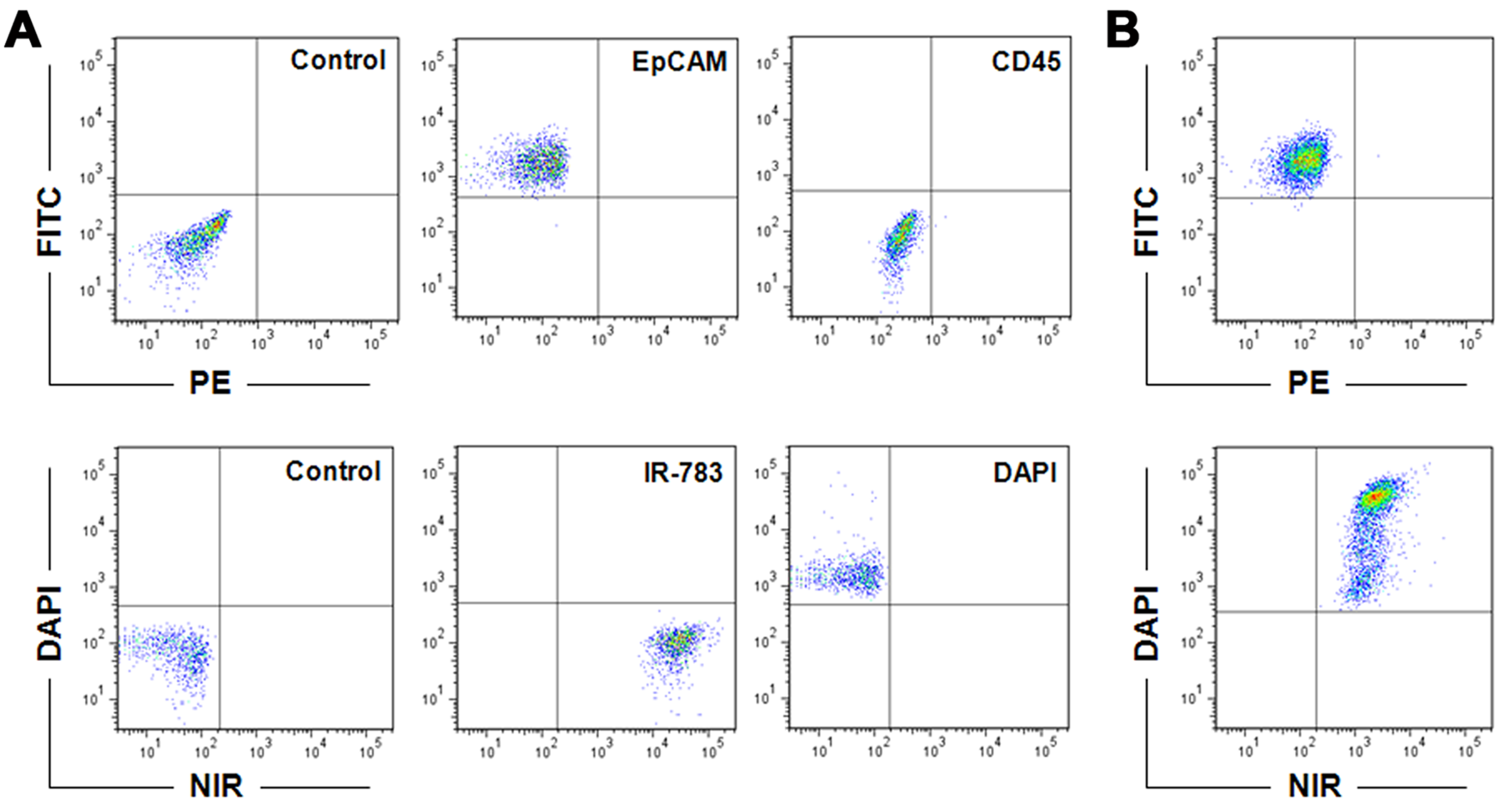

Supplement: Figure S1 — Confirmative staining of PC-3 cells with individual detection agents used in this study. A representative stain is shown. A, PC-3 cells stained with each individual detection agent were subjected to FACS analysis. In upper row, PC-3 cells stained by a mixture of mouse IgG1-FITC and IgG2b-PE were used as control. In the lower row, PC-3 cells in PBS were used as control. B, PC-3 cells were stained first with IR-783, then simultaneously with FITC-labeled antibody to EpCAM and PE-labeled antibody to CD45, and finally stained with DAPI. The stained cells were first detected for EpCAM expression and for CD45 exclusion (upper panel), and then for NIR and DAPI staining (power panel). (TIF) [file pone.0088967.s001.tif]

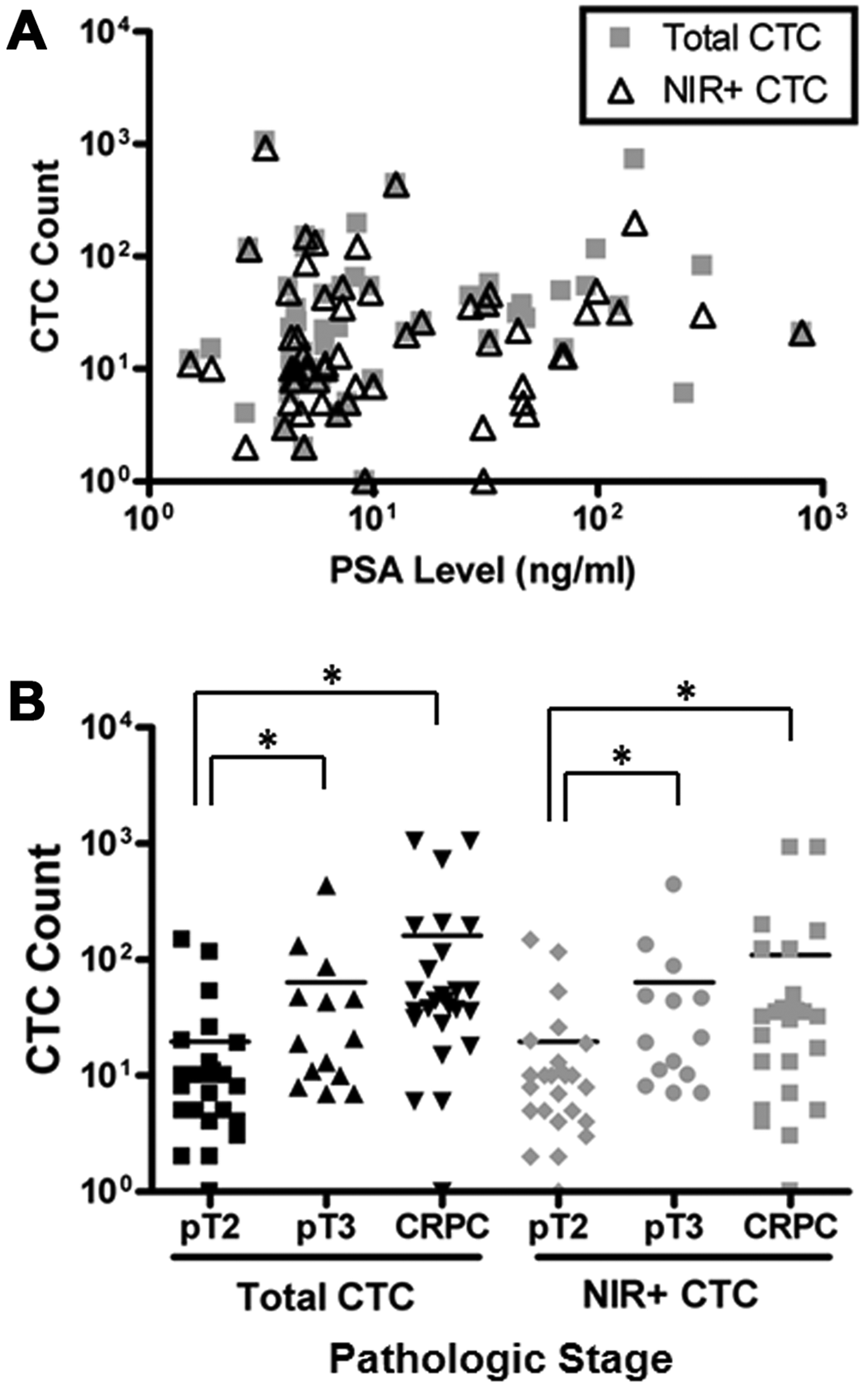

Supplement: Figure S2 — Lack of statistical significance when CTC counts from different patients were pooled for correlation analyses. Counts of the candidate CTCs from 40 primary prostate cancer patients and 23 samples from 5 mCRPC cases were analyzed against serum PSA level. (TIF) [file pone.0088967.s002.tif]

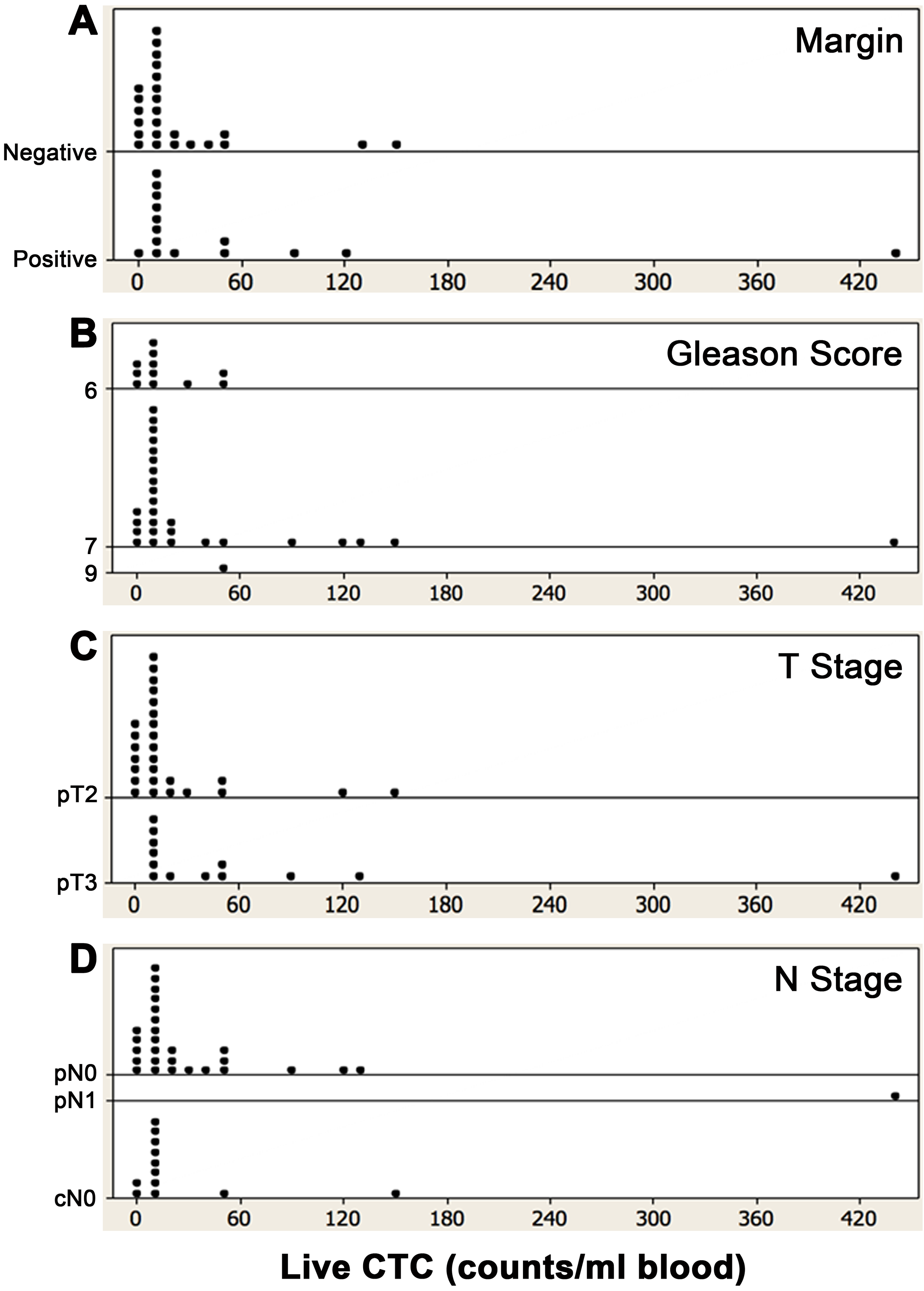

Supplement: Figure S3 — Lack of statistical significance between CTC counts and common diagnostic parameters. Counts of the candidate CTCs from 40 primary prostate cancer patients and 23 samples from 5 mCRPC cases were analyzed against surgical margin, Gleason score, T-stage and N-stage of the patient. (TIF) [file pone.0088967.s003.tif]

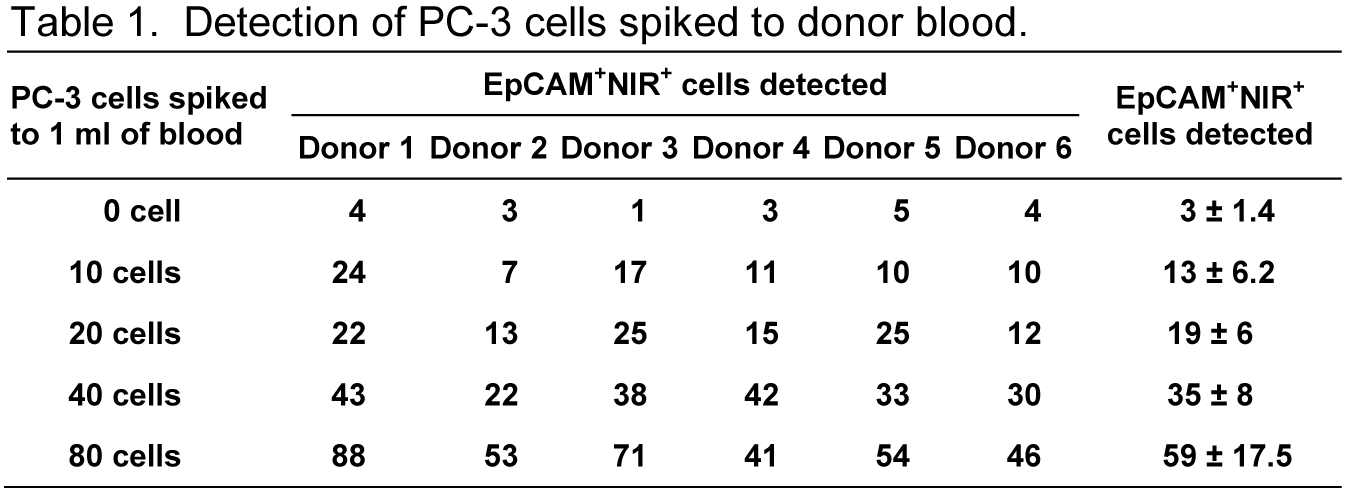

Supplement: Table S1 — Detection of PC-3 cells spiked to donor blood. (TIF) [file pone.0088967.s004.tif]
